# Can You Ride a Bicycle? The Ability to Ride a Bicycle Prevents Reduced Social Function in Older Adults With Mobility Limitation

**DOI:** 10.2188/jea.JE20150017

**Published:** 2016-06-05

**Authors:** Ryota Sakurai, Hisashi Kawai, Hideyo Yoshida, Taro Fukaya, Hiroyuki Suzuki, Hunkyung Kim, Hirohiko Hirano, Kazushige Ihara, Shuichi Obuchi, Yoshinori Fujiwara

**Affiliations:** 1 Faculty of Sport Sciences, Waseda University, Tokorozawa, Saitama, Japan; 1 早稲田大学 スポーツ科学学術院; 2 Research Team for Social Participation and Community Health, Tokyo Metropolitan Institute of Gerontology, Tokyo, Japan; 2 東京都健康長寿医療センター研究所 社会参加と地域保健研究チーム; 3 Research Fellow of the Japan Society for the Promotion of Science, Japan Society for the Promotion of Science, Tokyo, Japan; 3 日本学術振興会特別研究員; 4 Research Team for Human Care, Tokyo Metropolitan Institute of Gerontology, Tokyo, Japan; 4 東京都健康長寿医療センター研究所 福祉と生活ケア研究チーム; 5 Research Team for Promoting Independence of the Elderly, Tokyo Metropolitan Institute of Gerontology, Tokyo, Japan; 5 東京都健康長寿医療センター研究所 自立促進と介護予防研究チーム; 6 Department of Public Health, Toho University School of Medicine, Tokyo, Japan; 6 東邦大学 医学部 社会医学講座公衆衛生学分野

**Keywords:** older adults, cyclist, bicycling, mobility limitation, functional capacity, 高齢者, 自転車運転者, 自転車運転, 歩行制限, 手段的自立度

## Abstract

**Background:**

The health benefits of bicycling in older adults with mobility limitation (ML) are unclear. We investigated ML and functional capacity of older cyclists by evaluating their instrumental activities of daily living (IADL), intellectual activity, and social function.

**Methods:**

On the basis of interviews, 614 community-dwelling older adults (after excluding 63 participants who never cycled) were classified as cyclists with ML, cyclists without ML, non-cyclists with ML (who ceased bicycling due to physical difficulties), or non-cyclists without ML (who ceased bicycling for other reasons). A cyclist was defined as a person who cycled at least a few times per month, and ML was defined as difficulty walking 1 km or climbing stairs without using a handrail. Functional capacity and physical ability were evaluated by standardized tests.

**Results:**

Regular cycling was documented in 399 participants, and 74 of them (18.5%) had ML; among non-cyclists, 49 had ML, and 166 did not. Logistic regression analysis for evaluating the relationship between bicycling and functional capacity revealed that non-cyclists with ML were more likely to have reduced IADL and social function compared to cyclists with ML. However, logistic regression analysis also revealed that the risk of bicycle-related falls was significantly associated with ML among older cyclists.

**Conclusions:**

The ability and opportunity to bicycle may prevent reduced IADL and social function in older adults with ML, although older adults with ML have a higher risk of falls during bicycling. It is important to develop a safe environment for bicycling for older adults.

## Abstract (ja)

**【背景】:**

自転車利用が健康増進に寄与することは知られているが、歩行制限があるような高齢者においてもそのような恩恵があるか否かについては明らかではない。そこで本研究では、高次生活機能［手段的日常生活動作能力（以後、IADL）、知的能動性、社会的役割］に着目して、自転車利用が歩行制限のある高齢者に与える影響を明らかにすることを目的とした。

**【方法】:**

調査参加者のうち、自転車を運転したことの無い63名を除く614名を解析対象者とした。月に数回以上自転車に乗る高齢者を定期自転車運転者と定義し、1km歩き続けるもしくは手すりを使わず階段を上ることが難しい者を歩行制限者と定義した。高次生活機能は老研式活動能力指標を用いて測定した。

**【結果】:**

解析対象者のうち399名が定期自転車運転者であり、そのうち、74名（18.5%）が歩行制限を有していた。また、166名の非自転車運転者のうち、49名が歩行制限を有していた。交絡因子を調整したロジスティク回帰分析の結果、歩行制限のある非自転車運転者は歩行制限のある自転車運転者に比べてIADLと社会的役割が低下しやすいことが明らかとなった。他方で、歩行制限のある自転車運転者は歩行制限のない自転車運転者に比べて、自転車運転時の転倒の危険性が有意に高いことが明らかとなった。

**【結論】:**

本研究の結果から、歩行制限のある高齢者であっても、自転車を運転する能力や機会はIADLや社会的役割の維持に有効であることが分かった。しかしながら、歩行制限のある高齢者では自転車運転中に転倒しやすいことから、高齢者が自転車を運転しやすい環境づくりが重要であることが推察される。

## INTRODUCTION

**Figure 1.**
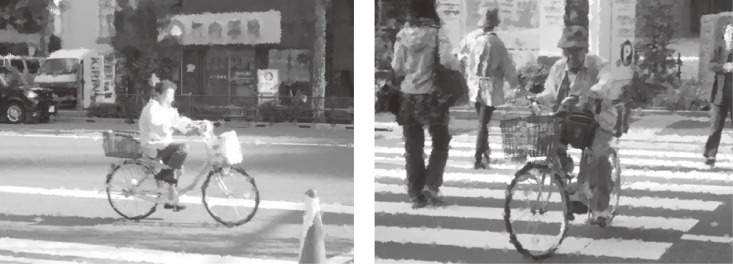
Elderly Japanese cyclists.

In many Asian countries, including Japan, bicycling is the most accessible mode of transportation. Interestingly, a recent large-scale mail survey indicated that 63% of community-dwelling Japanese older adults in urban areas routinely bicycle (Figure 1).^1^ As bicycling can provide health benefits,^2^^,^^3^ increasing the prevalence of bicycling among older adults might promote health maintenance. In addition, bicycling may also facilitate social interactions among older adults by providing an expanded range of available activities. This might be accentuated in urban-dwelling older adults because they rarely own cars. Therefore, the ability of older adults to ride a bicycle is likely correlated with their fine functional capacity, which is a crucial component of healthy independent living, even if they have mobility limitation (ML), such as difficulty walking or climbing stairs.^4^^,^^5^

In healthy older adults, functional capacity is operationally evaluated by three subscales: instrumental activities of daily living (IADL; instrumental self-maintenance), intellectual activity, and social function; these are based on Lawton’s model^6^ and categorize the stages of competence from the lowest/most basic function to the highest. Regarding IADL, difficulty walking is a significant predictor of reduced IADL.^7^ Furthermore, emerging evidence shows that healthy older adults are more likely to experience reduced intellectual activity and social function with advancing age.^8^^,^^9^ These findings suggest that IADL and intellectual activity/social function are vulnerable to ML and aging, respectively.

Although ML is associated with reduced IADL, older adults with ML who can ride a bicycle (cyclists with ML) may have a greater range of IADL than older adults with ML who cease bicycling (non-cyclists with ML), because the ability to ride a bicycle requires sufficient physical abilities (eg, muscle strength, balance, and flexibility) to ride stably with appropriate body and limb control. Older cyclists with ML likely have different physical abilities from non-cyclists with ML. Furthermore, bicycling provides access to friends, family, and public facilities, which can allow older adults to maintain their intellectual activity and social function by facilitating social interactions. Therefore, we hypothesize that the ability to ride a bicycle can prevent reduced functional capacity among older adults with ML.

Another relevant topic is whether bicycling among older adults with ML increases the incidence of bicycle-related falls. Compared to older cyclists without ML, older cyclists with ML may be more likely to lose their balance during bicycling because of physical difficulties, such as reduced balance and postural reflex. The present study investigated the following: (1) the prevalence of bicycling among older adults with ML; (2) the interaction effects between bicycling and the prevalence of ML for physical function; (3) whether the inability to ride a bicycle is associated with reduced functional capacity; (4) whether older cyclists with ML differ from older adults with ML who have ceased bicycling because of decreased physical ability; and (5) whether the prevalence of bicycle-related falls among older cyclists is associated with ML. To this end, we interviewed community-dwelling older adults and classified them as cyclists with or without ML or non-cyclists with or without ML and subsequently evaluated their functional capacity and physical ability using standardized tests.

## METHODS

### Participants

**Figure 2.**
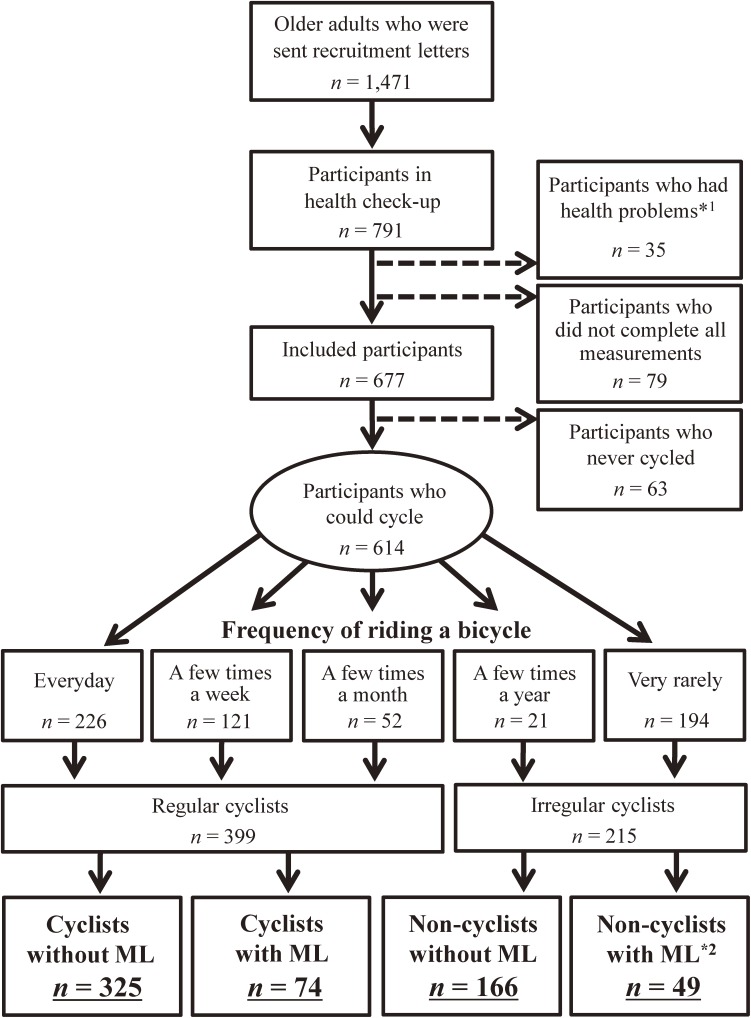
Schematic diagram of the study groups.  *^1^Participants who received additional assistance with their ADLs and had serious conditions or injuries (eg, depression, stroke, heart disease, and injury-related falls); *^2^Participants who ceased cycling owing to mobility limitation (physical difficulties); ML, mobility limitation.

Data were collected from a large health check-up held at the Tokyo Metropolitan Institute of Gerontology in 2013. On the basis of local resident registration, we mailed recruitment letters for the health check-up to 1471 older adults dwelling in an urban area (Itabashi Ward, Tokyo). A total of 791 community-dwelling older adults (mean age [standard deviation {SD}], 73.4 [5.0] years; range, 65–86 years; 57.0% women) participated in the health check-up. Participants who received additional assistance with their ADL, had serious conditions or injuries (eg, stroke, heart disease, and injury-related falls), or did not complete all measurements were excluded. Thus, 677 older adults with a mean (SD) age of 73.3 (5.6) years (range, 65–86 years; 58.0% women) were included (Figure 2). Written informed consent was obtained from all participants before the examination. The study was conducted in accordance with the ethical standards of the Declaration of Helsinki (2008),^10^ and the research protocol was approved by the Ethics Committee of the Tokyo Metropolitan Institute of Gerontology.

### Mobility limitation, bicycle-related information, functional capacity, and health-related background information

A trained interviewer investigated the participants’ ML, bicycling-related information, functional capacity, and health-related background information, including age, anamnesis (hypertension, cerebrovascular disorder, cardiac disease, diabetes mellitus, osteoporosis, and arthropathy), mood, and frequency of going outdoors.

ML was assessed by asking the participants whether they experienced difficulty walking 1 km, or climbing stairs without using a handrail.^4^^,^^5^ Participants reporting difficulty of either one were assigned to ML participants.

For bicycle-related information, participants were asked about the frequency of bicycling, with responses categorized as “every day”, “a few times a week”, “a few times a month”, “a few times a year”, or “very rarely”. The participants who responded “every day” to “a few times a month” and “a few times a year” or “very rarely” were categorized as regular and irregular cyclists, respectively (Figure 2). Irregular cyclists were then asked why they did not bicycle regularly. Those who ceased bicycling because of ML (ie, physical difficulties) were categorized as non-cyclists with ML, while those who ceased bicycling for other reasons were categorized as non-cyclists without ML.

Functional capacity was evaluated using the Tokyo Metropolitan Institute of Gerontology Index of Competence (TMIG-IC),^11^ which is a questionnaire that consists of three multidimensional subscales: IADL, intellectual activity, and social function. Three sublevels of competence were calculated: scores from 0 to 5 for IADL (able to use public transportation independently, shop for daily necessities, prepare meals independently, pay bills, and manage banking independently), scores from 0 to 4 for intellectual activity (able to fill out pension forms, read newspapers, and read books or magazines, and interest in news stories or TV programs that address health topics), and scores from 0 to 4 for social function (visiting friends at their homes, giving advice to family or a friend, able to visit sick friends, and speaking to young people). Higher scores indicate greater functional capacity.

Mood was evaluated using the 20-question Zung Self-Rating Depression Scale (SDS); scores range from 20 to 80, with higher scores indicating more severe depression.^12^ The participants’ daily routine for going outdoors was classified as high (go out every day) or low (go out every few days or less) frequency.^13^^,^^14^

### Physical ability and cognitive function

Physical ability was assessed by grip strength, one-leg standing, comfortable walking speed, the timed up and go (TUG) test, and leg extension strength.^5^^,^^15^^,^^16^ The maximum grip strength of the dominant hand was measured twice using a handheld Smedley-type dynamometer, and the highest value was recorded as the participant’s maximum grip strength. For the one-leg standing test, the participants were instructed to stand on their non-dominant leg, with their eyes open. Timing started when the participant’s dominant foot left the ground and stopped when the raised foot touched the ground again or when the participant successfully stood on one leg for 60 s. If the participant did not reach 60 s on the first attempt, a second attempt was permitted, and the longer time was recorded. To determine the participants’ comfortable walking speed, a flat 16-m walking path was marked with tape at the 3- and 13-m points. A stopwatch was used to measure the time taken to walk 10 m (ie, the time when a foot touched the ground past the 3-m line to when a foot touched the ground past the 13-m line). In the TUG test, participants were asked to stand up from a chair, briskly walk 3 m, turn around, and return to a seated position. The elapsed time for the TUG test was measured in seconds. The test was performed twice, and the shorter time was recorded. Leg extension strength was measured using a handheld dynamometer. Participants were asked to sit in a chair and perform a maximal isometric knee extension twice; the higher score was recorded.

Cognitive function was assessed by using the Mini-Mental State Examination (MMSE), which is a widely used tool for assessing overall cognitive function. The MMSE has a maximum score of 30 points, with higher scores indicating higher overall cognitive function.^17^

### Data analysis

Regarding interval variables, two-way analysis of variance (ANOVA) was performed for both bicycling (ie, cyclist vs non-cyclist) and ML (ie, ML vs non-ML) factors. In addition, differences in the categorical variables among cyclists with ML, cyclists without ML, non-cyclists with ML, and non-cyclists without ML were assessed using the χ^2^ test. Adjusted logistic regression analysis was performed to evaluate the relationship between bicycling and functional capacity. For this analysis, reductions in each functional capacity were defined as reported scores below the maximum (<5 for IADL and <4 for intellectual activity and social function) to set the functional capacity as the dependent variables. In this case, as there were multicollinearities in the physical abilities of the cyclist groups (*r* > 0.5), grip strength was used as a representative measure of physical ability as an independent variable. To examine the factors associated with the inability to ride a bicycle among older adults with ML, logistic regression analysis adjusted for health-related covariates was also performed for cyclists and non-cyclists with ML. Finally, to examine the relationship between ML and bicycle-related falls among older cyclists, logistic regression analysis was performed for cyclists with and without ML, adjusting for the frequency of bicycling and other covariates. All statistical analyses were performed using IBM SPSS Statistics, version 20.0 (SPSS Inc., Chicago, IL, USA), and a *P* value less than 0.05 was considered statistically significant.

## RESULTS

Participant enrollment is illustrated in Figure 2. Sixty-three participants had never ridden a bicycle and were excluded from analysis, and of the 399 participants who regularly cycled, 74 had ML (18.5% of cyclists). Among the 215 irregular cyclists, 49 had ceased bicycling due to physical difficulties (non-cyclists with ML), while the remaining 166 non-cyclists ceased bicycling for other reasons (eg, older adult who is easy to access to their destination using car, train, or others).

The participants’ characteristics are listed in Table 1. The results of two-way ANOVA of bicycling (ie, cyclists vs non-cyclists) and the presence of ML (ie, non-ML vs ML) are also shown in Table 1. The mixed-design two-way ANOVA revealed significant main effects of both bicycling and the presence of ML for all variables except intellectual activity and BMI (only for the main effects of ML). There were significant interactions between the two factors for IADL, comfortable walking speed, and the TUG (*P* = 0.005, *P* = 0.004, and *P* < 0.001, respectively). For IADL, post-hoc analyses showed significant simple main effects of group for non-cyclist and ML (*P* < 0.001). For comfortable walking speed and the TUG, there were significant simple main effects of cyclists, non-cyclists, ML, and non-ML (*P* < 0.001 for all). The χ^2^ test revealed that the percentage of women and having bicycle-related fall experience within the past year and the prevalences of hypertension, osteoporosis, and knee osteoarthritis differed significantly among the groups.

**Table 1.** Subject characteristics and mixed-design two-way ANOVA of bicycling (cyclists vs non-cyclists) and the presence of ML (non-ML vs ML)

Variables	Non-ML cyclists	ML cyclists	Non-ML non-cyclists	ML non-cyclists	*P*-value of ANOVA		
	(*n* = 325)	(*n* = 74)	(*n* = 166)	(*n* = 49)	Bicycling	ML	Interaction
Female, *n* (%)	170 (52.3)	46 (62.2)	83 (50.0)	35 (71.4)			0.024^b^
Age, years	71.7 (5.1)	74.6 (5.2)	73.9 (5.3)	77.1 (5.6)	*P* < 0.001	*P* < 0.001	0.794
Functional capacity: IADL	4.99 (0.11)	4.95 (0.23)	4.95 (0.24)	4.76 (0.62)	*P* < 0.001	*P* < 0.001	0.005
Functional capacity: Intellectual activity	3.82 (0.45)	3.65 (0.63)	3.77 (0.53)	3.71 (0.58)	0.872	0.032	0.274
Functional capacity: Social function	3.71 (0.61)	3.45 (1.03)	3.56 (0.70)	3.06 (1.13)	0.005	*P* < 0.001	0.356
BMI, kg/m^2^	22.7 (2.8)	23.6 (3.8)	22.7 (3.41)	23.4 (3.6)	0.789	0.012	0.745
MMSE	28.5 (1.8)	28.2 (1.7)	28.3 (2.1)	27.6 (2.1)	0.034	0.013	0.230
SDS	29.2 (5.5)	32.2 (8.2)	31.4 (8.0)	35.6 (9.3)	*P* < 0.001	*P* < 0.001	0.405
Grip strength, kg	30.7 (8.0)	27.1 (7.2)	29.2 (8.2)	23.8 (5.9)	0.004	*P* < 0.001	0.259
Comfortable gait speed, m/min	44.9 (6.1)	40.2 (6.0)	43.1 (5.9)	34.8 (7.1)	*P* < 0.001	*P* < 0.001	0.004
Timed Up & Go test, sec	5.3 (0.9)	6.2 (1.2)	5.7 (0.9)	7.9 (2.3)	*P* < 0.001	*P* < 0.001	*P* < 0.001
One-leg standing test, sec	48.5 (19.1)	32.4 (23.5)	41.9 (22.2)	19.9 (19.2)	*P* < 0.001	*P* < 0.001	0.166
Leg extension strength, N	314.9 (93.0)	274.7 (84.3)	293.2 (96.4)	232.2 (77.6)	0.001	*P* < 0.001	0.276
Low frequency of going outdoors^a^, *n* (%)	35 (10.8)	11 (14.9)	23 (13.9)	10 (20.4)			0.244^b^
Hypertension, *n* (%)	131 (40.3)	38 (51.4)	64 (38.6)	33 (67.3)			0.001^b^
Cerebrovascular disorder, *n* (%)	18 (5.5)	4 (5.4)	12 (7.2)	6 (12.2)			0.329^b^
Cardiac disease, *n* (%)	42 (12.9)	12 (16.2)	27 (16.3)	7 (14.3)			0.742^b^
Diabetes mellitus, *n* (%)	38 (11.7)	14 (18.9)	21 (12.7)	10 (20.4)			0.182^b^
Osteoporosis, *n* (%)	30 (9.2)	9 (12.2)	16 (9.6)	13 (26.5)			0.004^b^
Knee osteoarthritis, *n* (%)	24 (7.4)	9 (12.2)	11 (6.6)	12 (24.5)			0.001^b^
Bicycle-related Fall experience within a year, *n* (%)	21 (6.5)	15 (20.3)					*P* < 0.001^b^

Variables are reported as mean (standard deviation), unless otherwise noted.

BMI, body mass index; IADL, instrumental activities of daily living; ML, mobility limitation; MMSE, Mini-Mental State Examination; SDS, Zung Self-Rating Depression Scale.

^a^Older adults who went out once every 2–3 days or less.

^b^Results of the χ^2^ test.

Regarding functional capacity, 22 (3.6%), 112 (18.2%), and 180 (29.3%) older adults had reduced IADL (IADL score <5), reduced intellectual activity (intellectual activity score <4), and reduced social function (social function score <4), respectively. When the cyclists with ML were used as a reference, adjusted logistic regression analysis (Table 2) showed that reduced IADL and social function were significantly associated with only non-cyclists with ML (odds ratio [OR] 5.11, 95% confidence interval [CI] 1.12–23.22; and OR 2.33, 95% CI 1.06–5.13, respectively); intellectual activity was not associated with any cyclist group.

**Table 2.** Logistic regression analysis of disability in each functional capacity (*n* = 614)

Variables		IADL			Intellectual activity			Social function		
		
		OR	95% CI	*P*-value	OR	95% CI	*P*-value	OR	95% CI	*P*-value
Sex	Female (reference: male)	0.54	0.14–2.14	0.378	0.89	0.45–1.75	0.732	0.45	0.25–0.80	0.007
Age	Increment of 1 year of age	1.09	1.00–1.20	0.052	0.98	0.94–1.03	0.403	1.01	0.97–1.05	0.535
BMI	1 increment	0.90	0.76–1.07	0.247	1.06	0.99–1.14	0.114	0.99	0.93–1.05	0.700
MMSE	1 decrement	1.04	0.85–1.29	0.684	1.15	1.03–1.29	0.011	1.05	0.95–1.16	0.353
SDS	1 increment	1.02	0.96–1.07	0.586	1.05	1.01–1.08	0.004	1.08	1.05–1.11	*P* < 0.001
Grip strength	1 kg decrement	0.97	0.89–1.06	0.529	1.02	0.97–1.06	0.504	1.02	0.99–1.06	0.199
Frequency of going outdoors	Low frequency of going outdoors^a^ (reference: high frequency of going outdoors)	3.11	1.15–8.40	0.025	2.32	1.34–4.02	0.003	2.66	1.61–4.39	*P* < 0.001
Cyclist patterns	ML cyclists (reference)	1.00			1.00			1.00		
	ML non-cyclists	5.11	1.12–23.22	0.035	0.61	0.24–1.52	0.287	2.33	1.06–5.13	0.035
	Non-ML cyclists	0.33	0.07–1.67	0.181	0.62	0.33–1.19	0.155	0.72	0.40–1.32	0.288
	Non-ML non-cyclists	0.93	0.22–3.98	0.918	0.62	0.31–1.22	0.167	1.21	0.65–2.25	0.544

BMI, body mass index; CI, confidence interval; IADL, instrumental activities of daily living; ML, mobility limitation; MMSE, Mini-Mental State Examination; OR, odds ratio; SDS, Zung Self-Rating Depression Scale.

For independent variables, disability in each functional capacity was defined as a score below the maximum score (<5 for IADL and <4 for the intellectual activity and social function scales).

^a^Older adults who went out once every 2–3 days or less.

The results of logistic regression analysis examining the determinants of inability to ride a bicycle among older adults with ML are shown in Table 3. Only reduced TUG test time (OR 1.61; 95% CI, 1.02–2.60) was independently associated with the inability to ride a bicycle (ie, the decision to cease riding a bicycle) among older adults with ML.

**Table 3.** Logistic regression analysis of the inability to ride a bicycle among older adults with mobility limitations (*n* = 123)

Variables		OR	95% CI	*P*-value
Sex	Female (reference: male)	1.64	0.32–8.55	0.554
Age	Increment of 1 year of age	1.07	0.98–1.18	0.150
BMI	1 increment	0.96	0.82–1.13	0.621
MMSE	1 decrement	1.12	0.86–1.45	0.407
SDS	1 increment	1.03	0.98–1.09	0.278
Frequency of going outdoors	Low frequency of going outdoors^a^ (reference: high frequency of going outdoors)	0.99	0.28–3.51	0.990
Grip strength	1 kg decrement	1.00	0.89–1.13	0.953
Comfortable gait speed	1 sec/m decrement	1.02	0.93–1.12	0.712
Timed Up & Go test	1 sec increment	1.61	1.02–2.60	0.047
One-leg standing test	1 sec decrement	1.01	0.99–1.03	0.407
Leg extension strength	1 N decrement	1.00	0.99–1.01	0.897

BMI, body mass index; CI, confidence interval; MMSE, Mini-Mental State Examination; OR, odds ratio; SDS, Zung Self-Rating Depression Scale.

^a^Older adults who went out once every 2–3 days or less.

The results of logistic regression analysis of the relationship between ML and the risk of bicycle-related falls among cyclists are shown in Table 4. The risk of bicycle-related falls was independently associated with ML (OR 3.26; 95% CI, 1.37–7.76) as well as sex (OR 0.08; 95% CI, 0.02–0.29), SDS (OR 1.07; 95% CI, 1.02–1.13), and grip strength (OR 1.12; 95% CI, 1.03–1.21) among older adult cyclists.

**Table 4.** Logistic regression analysis of the risk factors for bicycle-related falls among older cyclists (*n* = 399)

Variables		OR	95% CI	*P*-value
Sex	Female (reference: male)	0.08	0.02–0.29	*P* < 0.001
Age	Increment of 1 year of age	0.99	0.91–1.07	0.712
BMI	1 increment	0.97	0.84–1.12	0.705
MMSE	1 decrement	0.80	0.61–1.04	0.091
SDS	1 increment	1.07	1.02–1.13	0.012
Grip strength	1 kg decrement	1.12	1.03–1.21	0.006
Frequency of going outdoors	Low frequency of going outdoors^a^ (reference: high frequency of going outdoors)	2.53	0.88–7.27	0.084
ML	ML cyclists (reference non-ML cyclists)	3.26	1.37–7.76	0.008
Frequency of bicycling	A few times a month (reference)	1.00		
	A few times a week	1.06	0.44–2.52	0.902
	Every day	0.30	0.07–1.34	0.115

BMI, body mass index; CI, confidence interval; ML, mobility limitation; MMSE, Mini-Mental State Examination; OR, odds ratio; SDS, Zung Self-Rating Depression Scale.

^a^Older adults who went out once every 2–3 days or less.

## DISCUSSION

The present study investigated the characteristics of older cyclists in Japan, focusing on mobility limitations and functional capacity. There are five key findings: (1) 18.5% of older cyclists had ML; (2) there was a significant interaction between bicycling and the prevalence of ML for IADL; (3) the ability to ride a bicycle was associated with the maintenance of IADL and social function among older adults with ML; (4) decreasing TUG test time was a predictor of the decision to cease riding a bicycle among older adults with ML; and (5) the risk of bicycle-related falls was significantly associated with ML among older cyclists. These findings suggest the ability and opportunity to ride a bicycle may contribute to the maintenance of functional capacity among older adults with ML, despite the increased risk of falling. As the prevalence of bicycling among community-dwelling older adults (≥65 years old) in Japan (63%)^1^ is reported to be much higher than in Western countries (7–26.2% [The upper value is the estimated average between men and women, as the previous study reports the rate of bicycling separately for each sex.]),^18^^–^^20^ the prevalence of bicycling among older adults with ML (18.5% of older cyclists) may be specific to Japanese older adults, particularly urban-dwellers.

Our results show that reduced IADL and social function were significantly associated with only non-cyclists with ML in comparison to cyclists with ML, suggesting that the ability to ride a bicycle is associated with the maintenance of IADL and social function among older adults with ML. This is partly supported by the results of the mixed-design two-way ANOVA, which showed a significant simple main effect of the ML group for the IADL. Although there are very few studies considering the ability to ride a bicycle as a part of IADL,^21^ the result of this study provides new insight that bicycling may contribute to maintenance of IADL even in those who have ML.

As mentioned above, we hypothesized that the ability to ride a bicycle is an important factor for preventing the reduction of functional capacity among older adults with ML. Our results are partially consistent with this hypothesis, as reduced IADL and social function were associated with non-cyclists with ML, although reduced intellectual activity was not. Since impaired intellectual activity and social function are reported to be significant predictors of future reductions of IADL,^8^^,^^9^ non-cyclists with ML might be more likely to experience reduced intellectual activity. Although it is unclear why only social function, but not intellectual activity, was independently associated with ML among non-cyclists, a likely explanation is that the decreasing trends in intellectual activity and social function differ with respect to area of residence. Previous studies have reported that social function is more likely to be reduced among urban-dwelling older adults, while intellectual activity is more likely to be reduced among rural-dwelling older adults.^8^^,^^9^ As our study’s participants lived in an urban area in Japan (Itabashi Ward, Tokyo), the association between reduced social function and non-cyclists with ML may have been more evident than that with intellectual activity.

Another possible explanation for the relationship between reduced social function and non-cyclists with ML is that bicycling provides access to acquaintances and public facilities, thereby allowing the participants to maintain their social function. The percentage of participants with a low frequency of going outdoors (ie, those who went outdoors every 2–3 days or less) in this study tended to be higher in those who did not cycle and in those who had ML (10.8% of cyclists without ML, 14.9% of cyclists with ML, 13.9% of non-cyclists without ML, and 20.4% of non-cyclists with ML). However, our results revealed that both of non-cyclists with ML and low frequency of going outdoors were independently associated with reduced social function. These results suggest the interaction between bicycling and ML (eg, non-cyclists with ML) for social function is not merely related to the frequency of going outdoors but rather to the quality of these trips. That is, the ability to visit friends who live far away regardless of the time of day might facilitate social interactions; such an increased opportunity to meet people may help prevent a reduction in social function.

Bicycling requires the ability to adequately control balance, posture, and pedaling. Thus, our finding that TUG ability (ie, TUG test time) was associated with the ability to ride a bicycle in older adults with ML seems reasonable, because TUG ability reflects multi-limb motor control ability, including the lower-extremity muscles and balance.^22^^–^^24^ Recent studies also suggest that TUG ability may be associated with cognitive function,^25^^,^^26^ particularly executive function (ie, inhibitory function and ability to multitask). The physical and cognitive abilities measured by the TUG test might regulate the ability of older adults with ML to ride a bicycle.

The risk of bicycle-related falls was significantly associated with ML among older cyclists. If cyclists come close to falling, they must quickly plant their feet on the ground to support their body. Compared to cyclists without ML, this ability to avoid a fall (ie, postural reflex) may be lower among cyclists with ML in addition to their decreased balance ability. It is well known that older adults with ML have poor physical function.^27^^,^^28^ Therefore, older adults with ML may be more likely to fall during bicycling than older adults without ML. Our findings indicate that, although bicycling is beneficial for maintaining functional capacity among older adults with ML, they have an increased risk of falls. These adults must take special care to confirm that current conditions are safe for bicycling (eg, riding safely and evaluating environmental features). The findings regarding the relationship between cyclists with ML and risk of falls also suggests that development and distribution of bicycles and other human-powered vehicles designed for fall prevention, such as tricycles, are important for the safety and comfort of older cyclists.

A potential limitation of this study is the use of a cross-sectional design, which precluded the determination of the causality of the relationships of the ability to ride a bicycle with ML and reduced functional capacity. However, the logistic regression analysis was controlled for several confounding factors that may have affected the participants’ functional capacity. It is important to investigate why social function was associated with the interaction between the ability to ride a bicycle and ML. Further longitudinal research is required to determine the causality of the relationships of the ability to ride a bicycle with ML and reduced functional capacity, as well as the mechanism(s) by which the ability to ride a bicycle affects social function among older adults with ML.

In conclusion, 18.5% of older cyclists had ML in our sample. Further, the ability to ride a bicycle was associated with the maintenance of IADL and social function among older adults with ML. However, although the ability to ride a bicycle may prevent reductions in IADL and social function among older adults, older cyclists with ML have a higher risk of falls during bicycling. Therefore, it is important to develop a safe environment for bicycling for older adults.

## ONLINE ONLY MATERIAL

Abstract in Japanese.
