# Supplementary material for: Can You Ride a Bicycle? The Ability to Ride a Bicycle Prevents Reduced Social Function in Older Adults With Mobility Limitation
Source: J Epidemiol. 2016 Jun 5;26(6):307–14. doi: 10.2188/jea.JE20150017 (PMC4884899; doi:10.2188/jea.JE20150017)
Supplement: Abstract in Japanese. [file je-26-307-s001.pdf]

自転車利用は歩行制限のある高齢者の社会的役割機能低下を防ぐ

桜井良太[1,2,3]、河合恒[4]、吉田英世[5]、深谷太郎[2]、鈴木宏幸[2]、金憲経[5]、平野浩彦[5]、井原一成[6]、大渕修一[4]、藤原佳典[2]

[1] 早稲田大学 スポーツ科学学術院
[2] 東京都健康長寿医療センター研究所 社会参加と地域保健研究チーム
[3] 日本学術振興会特別研究員
[4] 東京都健康長寿医療センター研究所　福祉と生活ケア研究チーム
[5] 東京都健康長寿医療センター研究所　自立促進と介護予防研究チーム
[6] 東邦大学 医学部 社会医学講座公衆衛生学分野

【背景】自転車利用が健康増進に寄与することは知られているが、歩行制限があるような高齢者においてもそのような恩恵があるか否かについては明らかではない。そこで本研究では、高次生活機能［手段的日常生活動作能力（以後、IADL）、知的能動性、社会的役割］に着目して、自転車利用が歩行制限のある高齢者に与える影響を明らかにすることを目的とした。

【方法】調査参加者のうち、自転車を運転したことの無い 63 名を除く 614 名を解析対象者とした。月に数回以上自転車に乗る高齢者を定期自転車運転者と定義し、1km 歩き続けるもしくは手すりを使わず階段を上ることが難しい者を歩行制限者と定義した。高次生活機能は老研式活動能力指標を用いて測定した。

【結果】解析対象者のうち 399 名が定期自転車運転者であり、そのうち、74 名（18.5%）が歩行制限を有していた。また、166 名の非自転車運転者のうち、49 名が歩行制限を有していた。交絡因子を調整したロジスティク回帰分析の結果、歩行制限のある非自転車運転者は歩行制限のある自転車運転者に比べて IADL と社会的役割が低下しやすいことが明らかとなった。他方で、歩行制限のある自転車運転者は歩行制限のない自転車運転者に比べて、自転車運転時の転倒の危険性が有意に高いことが明らかとなった。

【結論】本研究の結果から、歩行制限のある高齢者であっても、自転車を運転する能力や機会は IADL や社会的役割の維持に有効であることが分かった。しかしながら、歩行制限のある高齢者では自転車運転中に転倒しやすいことから、高齢者が自転車を運転しやすい環境づくりが重要であることが推察される。

キーワード: 高齢者、自転車運転者、自転車運転、歩行制限、手段的自立度
